# Classifying superconductivity in Moiré graphene superlattices

**DOI:** 10.1038/s41598-019-57055-w

**Published:** 2020-01-14

**Authors:** E. F. Talantsev, R. C. Mataira, W. P. Crump

**Affiliations:** 1 M.N. Miheev Institute of Metal Physics, Ural Branch, Russian Academy of Sciences, 18, S. Kovalevskoy St., Ekaterinburg, 620108 Russia; 2 NANOTECH Centre, Ural Federal University, 19 Mira St., Ekaterinburg, 620002 Russia; 3 Robinson Research Institute, Victoria University of Wellington, 69 Gracefield Road, Lower Hutt, 5040 New Zealand; 4 MacDiarmid Institute for Advanced Materials and Nanotechnology, P.O. Box 33436, Lower Hutt, 5046 New Zealand; 5 Aalto University, Foundation sr, PO Box 11000, FI-00076 AALTO, Finland

**Keywords:** Superconducting properties and materials, Electronic properties and devices, Superconducting properties and materials

## Abstract

Several research groups have reported on the observation of superconductivity in bilayer graphene structures where single atomic layers of graphene are stacked and then twisted at angles θ forming Moiré superlattices. The characterization of the superconducting state in these 2D materials is an ongoing task. Here we investigate the pairing symmetry of bilayer graphene Moiré superlattices twisted at θ = 1.05°, 1.10° and 1.16° for carrier doping states varied in the range of *n* = (0.5 − 1.5) · 10^12^ *cm*^−2^ (where superconductivity can be realized) by analyzing the temperature dependence of the upper critical field *B*_c2_(*T*) and the self-field critical current *J*_c_(sf,*T*) within currently available models – all of which start from phonon-mediated BCS theory – for single- and two-band *s*−, *d*−, *p*− an*d d* + *id-*wave gap symmetries. Extracted superconducting parameters show that only *s*-wave and a specific kind of *p*-wave symmetries are likely to be dominant in bilayer graphene Moiré superlattices. More experimental data is required to distinguish between the *s*- and remaining *p*-wave symmetries as well as the suspected two-band superconductivity in these 2D superlattices.

## Introduction

For an isotropic, spherical Fermi surface the density of states at the Fermi level is given by:

1 $$D({E}_{F})=\frac{8\pi }{{h}^{3}}\cdot {({m}^{\ast })}^{2}\cdot {v}_{F}$$

where *h* is the Planck constant, m^*^ is the effective mass of the charge carriers, and *v*_F_ is the Fermi velocity. Due to their large effective mass of m* ~ 200 · *m*_*e*_ (where *m*_e_ is the electron mass) heavy fermion superconductors^1^ possess a robust superconducting state, characterized by high values of the upper critical field *B*_c2_ ^2^ (significantly above the paramagnetic Pauli limit *B*_p_) despite a low Fermi velocity, ${v}_{F} \sim 5\cdot {10}^{3}\,m/s$, in these compounds^2^.

On the other hand, Eq. 1 prohibits a superconducting state in materials possessing a charge carrier effective mass of m* < 0.1 · *m*_*e*_. The is because a large value of *D*(*E*_F_) requires relativistic values for the Fermi velocity, ${v}_{F}\gtrsim {10}^{8}\,m/s$, which has not yet been observed in any material. In single layer graphene (SLG) and other materials with Dirac cone Fermi surfaces, *D*(*E*_F_) is given by:

2 $$D({E}_{F})=\frac{8\cdot \pi \cdot |{E}_{F}|}{{h}^{2}\cdot {v}_{F}^{2}},$$

which shows that *D*(*E*_F_) is inversely proportional to ${v}_{F}^{2}$. Therefore, the prerequisite to convert SLG and other planar honeycomb lattices^3^ into intrinsic superconductors is a reduction of *ν*_*F*_.

Lopes dos Santos *et al*.^4^ were the first to understand that *ν*_*F*_ can be suppressed in bilayer graphene by rotating the SLG sheets relative to each other by small twist angles θ. Detailed tight-binding model calculations performed by Bistritzer and MacDonald^5^ showed that at the Dirac points *ν*_*F*_ goes to zero when bilayer graphene is rotated by small angles, creating a Moiré superlattice. These graphene 2D structures are now called magic-angle twisted bilayer graphene (MATBG).

The discovery of intrinsic superconductivity in few-layer stanene (one of the closest analogues of graphene) which possesses a Fermi velocity of ${v}_{F}=4.5\cdot {10}^{4}\,m/s$ by Liao *et al*.^6^ gave clear experimental proof that the suppression of *v*_F_ in Dirac cone materials makes it possible to convert these materials into intrinsic superconductors. Several months after Liao’s work^6^, intrinsic superconductivity in MATBG with ${v}_{F} \sim 2\cdot {10}^{4}\,m/s$ and ${m}^{\ast } \sim 0.2\cdot {m}_{e}$ was reported by Cao *et al*.^7^. There, simultaneous *v*_F_ suppression and m* enhancement was achieved in the way considered by Lopes dos Santos *et al*.^4^ as well as Bistritzer and MacDonald^5^, i.e. by rotation of 2 stacked SLG sheets to form a Moiré superlattice. For instance, Cao *et al*.^7^ showed that MATBG with *θ* = 1.16° exhibits zero resistance at *T*_c_ = 0.14 K, whereas an angle *θ* = 1.05° has zero resistance at *T*_c_ ~ 1.2 K. More recently, several research groups have discovered a superconducting state in MATBG^8–11^, including studies of MATBG at high-pressures (up to *P* = 4 GPa), as well as in trilayer graphene/boron nitride Moiré superlattices^12^.

There are still many important questions regarding the superconducting state of MATBG that remain unanswered. In particular, the pairing mechanism and symmetry, as well as the number of superconducting bands, is not clear. While Cao *et al*.^7^ propose strong electron-electron correlations as the mechanism of the superconductivity, a full analysis with respect to existing theories for phonon mediation is still required. When treated as a phonon mediated superconductor the possibility of multiple superconducting bands arises from the fact that the charge carriers in MATBG experience two different phonon modes, one from intralayer covalent bonds, and a second mode due to the interlayer coupling (details can be found in^13,14^).

Here, we analyse temperature dependent measurements of the upper critical field and self-field critical current in bilayer twisted SLG structures reported by Cao *et al*.^7^ and Lu *et al*.^11^. This analysis is done in the existing phenomenology developed for phonon-mediated superconductors, to test if experimental data necessitates the development of a new phenomenology. We investigate *s−*, *d−*, *p−* and *d* + *id* pairing symmetry scenarios within single and two-band models. As a result, we found that phonon mediated *d-*wave, *d* + *id*-wave and three of the four *p*-wave pairing symmetries should be excluded from further consideration. More experimental data is required to confirm which of the remaining two (i.e., *s-*wave or axial *A*⊥*l p-*wave) pairing scenarios this material exhibits. As graphene has a planar honeycomb lattice of *sp*^2^ bonded carbon atoms, it is unsurprising that phonon-mediated superconductivity would exhibit pairing symmetry of either *s*- or *p*-wave in MATBG.

## Results

### Upper critical field analysis: single superconducting band models

We analyse the temperature dependent perpendicular upper critical field *B*_c2,⊥_(*T*) measured by Cao *et al*.^7^ [Fig. 3e] where the magnetic field is applied in the perpendicular direction to the MATBG plane. First we present the standard literature analysis for *B*_c2_(*T*) data by fitting the data to the 5 available models for *B*_c2,⊥_(*T*) and extracting *T*_c_ as well as the *ξ*(0). However, we extend this analysis by noting that the first 5 models presented have an implicit assumption of the behaviour of the penetration depth *λ*(*T*). By dropping this assumption, we can express *B*_c2,⊥_(*T*) as a function of the band gap Δ(*T*) and its symmetry.

**Figure 1:**
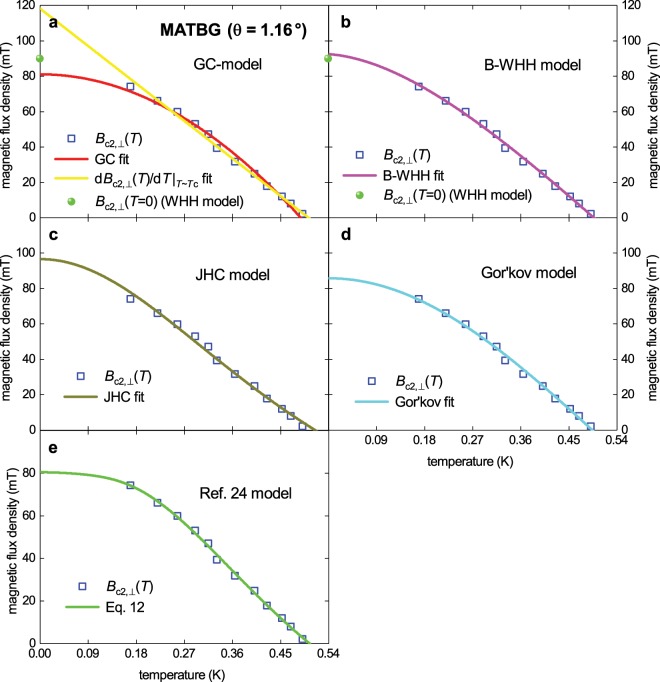
The upper critical field data, *B*_c2,⊥_(*T*), for sample M1 (*θ* = 1.16°) of measured by Cao *et al*.^7^ (squares) fitted to single band models. (**a**) red: fit to Gorter-Casimir (GC) two fluid model (Eq. 1) with a goodness of fit *R* = 0.9892 (*T*_c_ = 0.489 ± 0.004 K, *B*_c2,⊥_(0) = 81 ± 2 mT, ξ_ab_(0) = 64 ± 1 nm); yellow: Werthamer-Helfand-Hohenberg (WHH) model (Eq. 2), *R* = 0.9976 (*T*_c_ = 0.505 ± 0.005 K, *B*_c2,⊥_(0) = 81 ± 2 mT, ξ_ab_(0) = 64 ± 1 nm). (**b**) Baumgartner-WHH (B-WHH) model (Eq. 3 and 4), *R* = 0.9966 (*T*_c_ = 0.497 ± 0.003 K, *B*_c2,⊥_(0) = 92 ± 1 mT, ξ_ab_(0) = 60 ± 1 nm). (**c**) Jones-Hulm-Chandrasekhar (JHC) model Eq. 5, *R* = 0.9930 (*T*_c_ = 0.515 ± 0.005 K, *B*_c2,⊥_(0) = 97 ± 2 mT, ξ_ab_(0) = 58 ± 1 nm). (**d**) Gor’kov model Eq. 7, *R* = 0.9959 (*T*_c_ = 0.494 ± 0.003 K, *B*_c2,⊥_(0) = 86 ± 1 mT, ξ_ab_(0) = 62 ± 1 nm). (**e**) Model proposed in ref. ^24^, *R* = 0.9979 (*T*_c_ = 0.504 ± 0.007 K, *B*_c2,⊥_(0) = 80 ± 2 mT, ξ_ab_(0) = 64 ± 1 nm, Δ(0) = 81 ± 6 meV, Δ*C*/*C* = 1.46 ± 0.28, 2Δ(0)/*k*_B_*T*_c_ = 3.73 ± 0.28).

To begin, we fit the *B*_c2,⊥_(*T*) data to the Gorter-Casimir (GC) two fluid model^15,16^ which is still in wide use^17,18^:

3 $${B}_{c2,\perp }(T)=\frac{{\varphi }_{0}}{2\cdot \pi \cdot {\xi }_{ab}^{2}(0)}\cdot (1-{(\frac{T}{{T}_{c}})}^{2}),$$

where ${\varphi }_{0}=\frac{h}{2\cdot e}=2.067\cdot {10}^{-15}$ Wb is the superconducting flux quantum; composed of Plank’s constant *h* and the electron charge *e* is, and ξ_ab_(*T*) is the coherence length in the a-b plane. The fit is shown in Fig. 1(a).

Another widely used model for fitting *B*_c2_(*T*) data is the Werthamer-Helfand-Hohenberg (WHH) model^19,20^:

4 $${B}_{c2,\perp }(0)=-\,0.697\cdot {T}_{c}\cdot {(\frac{d{B}_{c2}(T)}{dT})}_{T \sim {T}_{c}},$$

a fit of the data to this model is also shown in Fig. 1(a). Baumgartner *et al*.^21^ proposed a simple and accurate analytical expression that matches the shape of the WHH model:

5 $${B}_{c2,\perp }(T)=\frac{1}{0.697}\cdot \frac{{\varphi }_{0}}{2\cdot \pi \cdot {\xi }_{ab}^{2}(0)}\cdot ((1-\frac{T}{{T}_{c}})-0.153\cdot {(1-\frac{T}{{T}_{c}})}^{2}-0.152\cdot {(1-\frac{T}{{T}_{c}})}^{4}).$$

This model will be designated as B-WHH and its fit of the data are shown in Fig. 1(b), where both values of *B*_c2,⊥_(*T* = 0 K) (i.e., original WHH and B-WHH) agree as expected.

Jones-Hulm-Chandrasekhar (JHC) proposed three models^22^, two of which are often used to fit *B*_c2_(*T*) data^23–25^. The first is a combination of Ginzburg-Landau (GL) theory with the Gorter-Casimir expression for the temperature dependence of *λ*(*T*):

6 $${B}_{c2,\perp }(T)=\frac{{\varphi }_{0}}{2\cdot \pi \cdot {\xi }_{ab}^{2}(0)}\cdot (\frac{1-{(\frac{T}{{T}_{c}})}^{2}}{1+{(\frac{T}{{T}_{c}})}^{2}}),$$

which we shall refer to as the JHC model, a fit using this model is shown in Fig. 1(c). This model gives a little higher value for *B*_c2,⊥_(*T* = 0 K) and *T*_c_ in comparison with the other models, as well as the lowest value of *ξ*_ab_ (0). The second model proposed by JHC is based on an expression from Gor’kov for *B*_c2_(*T*)^26^:

7 $${B}_{c2}(T)={B}_{c}(T)\cdot \frac{\sqrt{2}}{1.77}\cdot \frac{\lambda (0)}{\xi (0)}\cdot (1.77-0.43\cdot {(\frac{T}{{T}_{c}})}^{2}+0.07\cdot {(\frac{T}{{T}_{c}})}^{4}),$$

where *B*_c_(*T*) is the thermodynamic critical field, and λ(0) is the ground state London penetration depth. Jones *et al*.^22^ again use the Gorter-Casimir form of *λ*(*T*) to produce a model of the following form:

8 $${B}_{c2,\perp }(T)=\frac{1}{1.77}\cdot \frac{{\varphi }_{0}}{2\cdot \pi \cdot {\xi }_{ab}^{2}(0)}\cdot (1.77-0.43\cdot {(\frac{T}{{T}_{c}})}^{2}+0.07\cdot {(\frac{T}{{T}_{c}})}^{4})\cdot (1-{(\frac{T}{{T}_{c}})}^{2}).$$

Equation 8 will be referred to as the Gor’kov model.

However, rather than assume the behavior of the penetration depth, Eq. 7 can be used to build a model based directly on the BCS expression^16,27^ for *λ*(*T*) as a function of the superconducting gap:

9 $$\lambda (T)=\frac{\lambda (0)}{\sqrt{1-\frac{1}{2\cdot {k}_{B}\cdot T}\cdot {\int }_{0}^{\infty }\frac{d\varepsilon }{{\cosh }^{2}(\frac{\sqrt{{\varepsilon }^{2}+{\Delta }^{2}(T)}}{2\cdot {k}_{B}\cdot T})}}},$$

where Δ(*T*) is the temperature-dependent superconducting gap. Equation 9 captures the effect of the gap symmetry on *B*_c2_(*T*). An expression for Δ(*T*) as a function of the wavefunction symmetry is given by Gross-Alltag *et al*.^28^. For now, we test s-wave symmetry where the gap takes the form:

10 $$\Delta (T)=\Delta (0)\cdot \,\tanh [\frac{\pi \cdot {k}_{B}\cdot {T}_{c}}{\Delta (0)}\cdot \sqrt{\eta \cdot \frac{\Delta C}{C}\cdot (\frac{{T}_{c}}{T}-1)}],$$

where Δ*C*/*C* is the relative jump in electronic specific heat at *T*_c_, and *η* = 2/3. We can then use the standard GL expression:

11 $${B}_{c2}(T)=\sqrt{2}\cdot \frac{\lambda (T)}{\xi (T)}\cdot {B}_{c}(T),$$

to restate Gor’kov’s expression, Eq. 7, as:

12 $$\kappa (T)=\frac{\lambda (T)}{\xi (T)}=\frac{1}{1.77}\cdot \frac{\lambda (0)}{\xi (0)}\cdot (1.77-0.43\cdot {(\frac{T}{{T}_{c}})}^{2}+0.07\cdot {(\frac{T}{{T}_{c}})}^{4}).$$

Then, by considering another GL expression:

13 $${B}_{c2}(T)=2\cdot {(\frac{\lambda (T)}{\xi (T)})}^{2}\cdot \frac{{B}_{c1}(T)}{\mathrm{ln}(\kappa (T))+0.5}={(\frac{\lambda (T)}{\xi (T)})}^{2}\cdot \frac{{\varphi }_{0}}{2\cdot \pi \cdot {\lambda }^{2}(T)}={(\kappa (T))}^{2}\cdot \frac{{\varphi }_{0}}{2\cdot \pi \cdot {\lambda }^{2}(T)},$$

we can combine Eqs. (9–12) and Eq. 13 into a single expression for the temperature dependent upper critical field:

14 $$\begin{array}{rcl}{B}_{c2,\perp }(T) & = & \frac{{\varphi }_{0}}{2\cdot \pi \cdot {\xi }_{ab}^{2}(0)}\cdot {(\frac{1.77-0.43\cdot {(\frac{T}{{T}_{c}})}^{2}+0.07\cdot {(\frac{T}{{T}_{c}})}^{4}}{1.77})}^{2}\\  &  & \cdot \,[1-\frac{1}{2\cdot {k}_{B}\cdot T}\cdot {\int }_{0}^{\infty }\frac{d\varepsilon }{cos{h}^{2}(\frac{\sqrt{{\varepsilon }^{2}+{\Delta }^{2}(T)}}{2\cdot {k}_{B}\cdot T})}].\end{array}$$

Equation 14 is a function parameterized by four fundamental superconducting parameters, ξ_ab_(0), Δ(0), Δ*C*/*C*, and *T*_c_. It is critical to note that the role each parameter plays in the equation is well founded in BCS and GL theory. In particular ξ_ab_(0) determines the absolute value of *B*_c2,⊥_(0), independently of the other parameters. Furthermore, *T*_c_ is tightly constrained to define a reduced temperature scale *t* = *T/T*_c_, over which Gor’kov’s expression, Eq. 7, is valid. The only new feature of Eq. 14 is the introduction of the gap symmetry expression for *λ*(*T*), Eq. 9. This introduction, and that of the Gross-Alltag *et al*. expression for the gap Δ(*T*) itself, only introduces degrees of freedom, Δ(0) and Δ*C*/*C*, whose behavior were phenomenologically assumed by the literature models. The advantage of this method is that the two new parameters can be deduced and explicitly checked against the bounds of the theory’s validity.

A fit of the MATBG *B*_c2_(*T*) data to Eq. 14 is shown in Fig. 1(e) where the superconducting gap ratio was found to be:

15 $$\frac{2\cdot \Delta (0)}{{k}_{B}\cdot {T}_{c}}=3.73\pm 0.28$$

and the relative jump in electronic specific heat:

16 $${\frac{\Delta C}{C}|}_{T \sim {T}_{c}}=1.46\pm 0.28.$$

These deduced parameters, within their uncertainties, are remarkably close to the BCS weak-coupling limits of $\frac{2\cdot \Delta (0)}{{k}_{B}\cdot {T}_{c}}=3.53$ and ${\frac{\Delta C}{C}|}_{T \sim {T}_{c}}=1.43$. This is the first quantitative evidence that *intrinsic superconductivity* in MATBG can be understood in the existing phenomenology of *s*-wave electron-phonon mediated superconductivity and a new phenomenology may not need to be developed. Overall, the deduced values for ξ_ab_(0) and *T*_c_ using the six models are (see for details Supplementary Table S1): ${\xi }_{ab}(0)=61.4\pm 1.7\,nm$ ${T}_{c}=0.500\pm 0.006\,K$.

Once a value of the ground state gap has been obtained another standard BCS expression^16,27^ can be used to calculate *v*_F_ for the sample M1 (*θ* = 1.16°) based on the deduced values of ξ_ab_(0) and Δ(0):

17 $${v}_{F,ab}=\frac{2\cdot {\pi }^{2}\cdot \Delta (0)\cdot {\xi }_{ab}(0)}{h}=(2.37\pm 0.18)\cdot {10}^{4}\,m/s$$

The value given in Eq. 17 is about two orders of magnitude lower than the Fermi velocity of pure SLG (${v}_{F,ab} \sim {10}^{6}\,m/s$)^29^.

The Fermi temperature, *T*_F_, can be calculated in the usual way:

18 $${T}_{F}=\frac{{E}_{F}}{{k}_{B}}=\frac{{m}^{\ast }\cdot {v}_{F}^{2}}{2\cdot {k}_{B}},$$

where m^*^ is the effective mass of charge carriers. In the original paper by Cao *et al*.^7^, m^*^ was not measured for sample M1 (*θ* = 1.16°). A simple assumption is that at the same doping, MATBG with different *θ* should have the same effective mass, m*. By assuming this, and using *B*_c2_(*T*) as an indication of the doping state we can assume that for sample M1:

19 $${m}^{\ast }(n\, \sim -\,1.5\cdot {10}^{12}c{m}^{-2})\approx 0.2\cdot {m}_{e}.$$

(we present full analysis for this value for sample M2 in the following section). Corresponding to this m* value the Fermi temperature, *T*_F_, is:

20 $${T}_{F}=3.7\pm 0.5\,K$$

**Figure 2:**
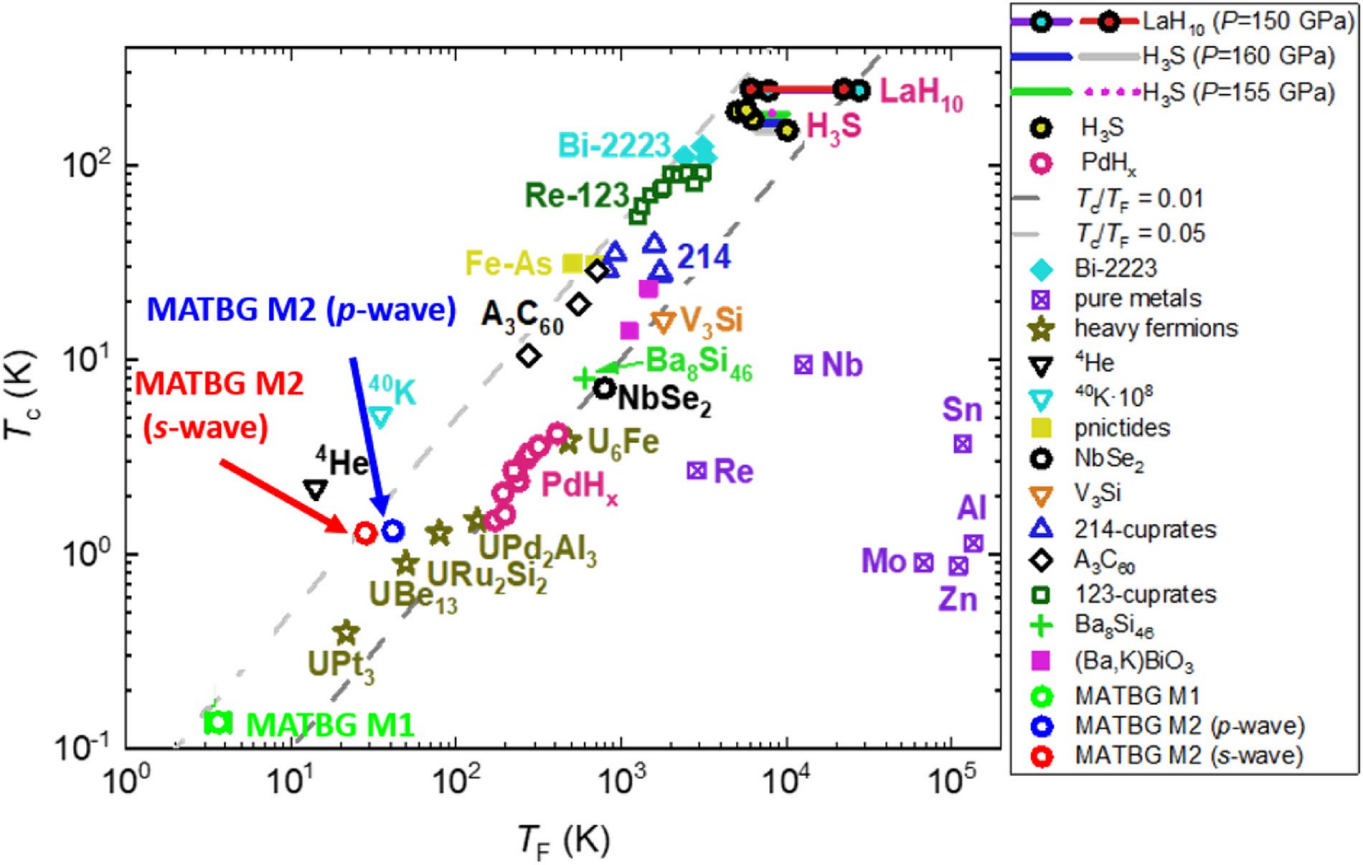
A plot of *T*_c_ versus *T*_F_ obtained for materials representative of the various superconducting families. Data was taken from Uemura^28^, Cao *et al*.^7^, as well as^24,25^.

We plot this data point for MATBG sample M1 in an Uemura plot^30^ (Fig. 2) where the transition temperature *T*_c_ is defined as the temperature at which order parameter phase coherence is established i.e. when the sample resistance becomes zero, *R* = 0 Ω (rather than R = 0.5R_n_). This definition is in accordance with all other data points (for other materials) and follows the general logic of the Uemura *et al*.^30,31^ definition of the transition temperature.

From *R*(*T*) data for sample M1 presented in^7^ [Fig. 1b] we determine the transition temperature to be *T*_c_ = 0.136 K. One therefore obtains:

21 $$\frac{{T}_{c}}{{T}_{F}}=0.037\pm 0.005 < 0.05,$$

which fits within the boundary of:

22 $$0.01\le \frac{{T}_{c}}{{T}_{F}} < 0.05,$$

established by Uemura *et al*.^30,31^ for all other unconventional superconductors.

Unfortunately, the existing data set is insufficient to reveal irreconcilable differences between the models analyzed. While this is reasonable considering how low the temperature must be taken, it is also unfortunate that *B*_c2,⊥_(*T*) data is not available for sample M2; where the higher critical temperature would lead to a lower acheivable range of reduced temperature *t* = *T*/*T*_c_.

It is possible to fit the *B*_c2,⊥_(*T*) data to a model with *d−*, *p*− and *d* + *id*-gap symmetry by substituting the relevant expressions for λ(*T*) and Δ(*T*) in Eq. 14, however, the *B*_c2,⊥_(*T*) data for the sample M1^7^ was not measured to low enough temperatures (*T*/*T*_c_ < 0.33) to accurately determine the fit parameters.

To resolve this issue we look at self-field critical current density data *J*_c_(sf, *T*) which was measured by Cao *et al*.^7^ for the sample M2 (*θ* = 1.05°) down to *T* = 0.05 K. These measurements, taking into account that *T*_c_ ~ 1.2 K, were taken down to a reduced temperature of *T*/*T*_c_ = 0.04 ≪ 0.33. Therefore, the raw *J*_c_(sf,*T*) data provides a meaningful insight into the superconducting gap symmetry of MATBG, which we will explore in a later section.

### Upper critical field analysis: two superconducting band model

As we already mentioned above, several authors have proposed a two-band superconducting state in MATBG which originates from two different phonon modes. One is due to intralayer covalent bonds, and the other is due to interlayer coupling (an extended reference list and discussion can be found in refs. ^13,14^). It is interesting to note that in our previous papers^17,32–34^ we found that many atomically thin superconductors, including SLG and topological insulators (with a proximity induced superconducting state) have a two-superconducting band state.

**Figure 3:**
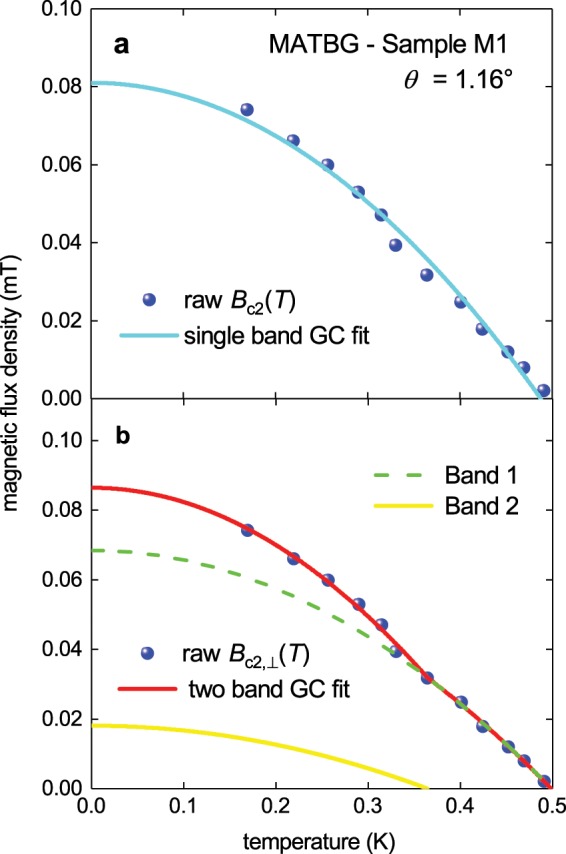
The upper critical field data, *B*_c2,⊥_(*T*), for sample M1 (*θ* = 1.16°) of measured by Cao *et al*.^7^ (balls) fitted to two GC two-band models. (**a**) fit to single band GC model (Eq. 1) with a goodness of fit *R* = 0.9892 (*T*_c_ = 0.489 ± 0.004 K, *B*_c2,⊥_(0) = 81 ± 2 mT, ξ_ab_(0) = 64 ± 1 nm); (**b**) fit to two-band GC model (Eq. 23)^17^, *R* = 0.9984 (*T*_c1_ = 0.498 ± 0.004 K, *B*_c2,⊥,1_(0) = 68 ± 4 mT, ξ_ab,1_(0) = 69.4 ± 1.8 nm, *T*_c2_ = 0.364 ± 0.016 K, *B*_c2,⊥,2_(0) = 18 ± 4 mT, ξ_ab,2_(0) = 135 ± 14 nm).

Looking closer at the upper critical field, *B*_c2,⊥_(*T*), fit to the single band GC model in Fig. 3(a), it can be seen that in the temperature range of *T* = 0.3–0.4 K the experimental *B*_c2,⊥_(*T*) data is well below the fitted curve. A fit of the data to a decoupled-two-band GC model (which we proposed in ref. ^17^):

23 $${B}_{c2,\perp }(T)={B}_{c2,\perp ,Band1}(T)+{B}_{c2,\perp ,Band1}(T),$$

is shown in Fig. 3(b). The deduced parameters, see Fig. 3(b), agree well with the mutual parameter interdependence criteria, which remains low and varies from 0.69 to 0.97 (the definition of this parameter can be found elsewhere^17^).

It would be interesting to analyse the experimental *B*_c2,⊥_(*T*) data using the models in the previous section, however this requires a more comprehensive raw *B*_c2,⊥_(*T*) data file which densely covers the reduced temperature range of the lower band (band 2) *t*_2_ = *T/T*_c2_ down to a level *t* <<0.3. This experimental data is not yet available.

### Self-field critical current analysis: single superconducting band models

The voltage-current, *V*(*I*), curves for sample M2 can be found in^7^ [Fig. 1e]. To extract *I*_c_(sf,*T*) the experimental *V*(*I*) curves are fitted by the standard power-law expression^35–37^:

24 $$V(I)={V}_{0}+k\cdot I+{V}_{c}\cdot {(\frac{I}{{I}_{c}})}^{n}\,$$

where *V*_0_ is an instrumental offset, *k* is a linear term used to accommodate incomplete current transfer in short samples, *n* is the flux creep exponent, and *V*_c_ is the voltage criteria which was chosen to be 10 μV. The superconducting state can be defined through the *V*(*I*) fit to Eq. 24 by a *n* > 1 criterion, where the normal state corresponds to *n* = 1 (Ohm’s law). Physically speaking, this approach defines detection of the superconducting phase coherence by detection of flux vortex flow in the sample. This distinction is necessary as data in Cao *et al*. shows an elongated transition from the normal to superconducting state^7^ [Fig. 1b]. Furthermore, this definition of *T*_c_ is in full accordance with accepted standard in the field^38–43^.

We find that *n*(*T* = 1.07 K) = 2.0 ± 0.2, where as *n*(*T* = 1.26 K) = 1.1 ± 0.7. The bridge resistance is found to be *R*(*T* = 1.26 K) = 1.56 kΩ, with a material resistivity *ρ*(*T* = 1.26 K) = 7.5 · 10^−7^ Ω·m (the MATBG thickness, 2*b*, was assumed to be 1.0 nm, i.e. around double the lower limit of the most accurate measurements of single layer graphene^44^, *d* = 0.43–1.69 nm). By Eq. 24, the highest temperature at which the superconducting coherence was confirmed in the experiment is *T* = 1.07 K (see, Figs. S1–S3, and Table S2).

**Figure 4:**
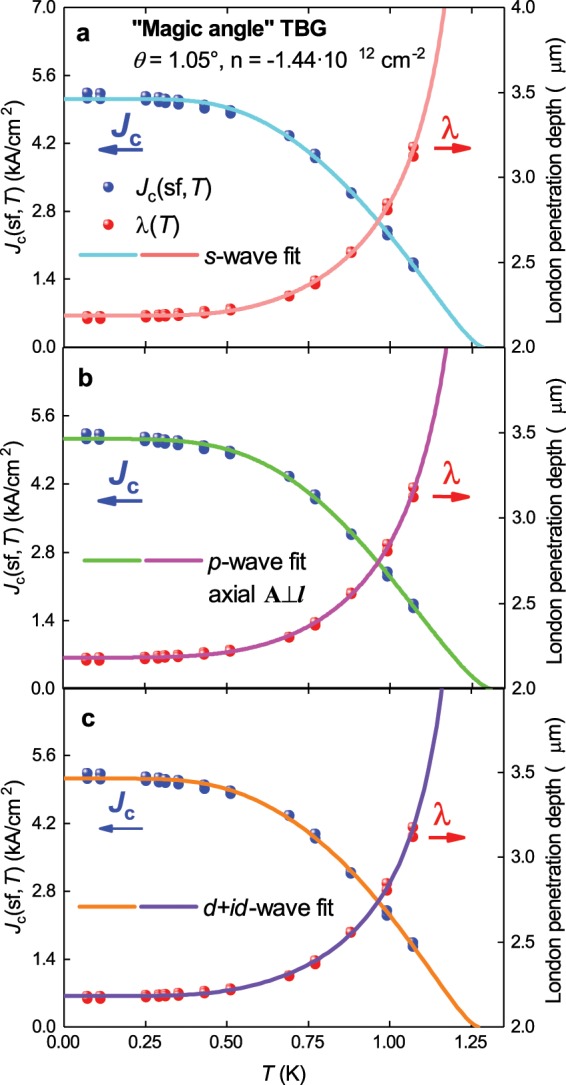
The self-field critical current density, *J*_c_(sf,*T*), for sample M2 (*θ* = 1.05°) from the work of Cao *et al*.^7^ and a fit of the data to three single-band models. For all models ξ_ab_(0) = 61.4 nm was used. (**a**) *s*-wave fit, the goodness of fit *R* = 0.9964; (**b**) *p*-wave axial **A**⊥***l*** fit, *R* = 0.9971; (**c**) *d* + *id*-wave fit, *R* = 0.9967. Deduced parameters are listed in Table 1.

The resulting *J*_c_(sf,*T*) = *I*_c_(sf,*T*)/(4ab) (where 2*a* is the sample M2 width, 2*a* = 1.05 μm) is shown in Fig. 4. In this figure the *J*_c_(sf,*T*) data was fitted using different superconducting gap symmetries by the equation^45^:

25 $${J}_{c}(sf,T)=\frac{{\varphi }_{0}}{4\cdot \pi \cdot {\mu }_{0}}\cdot \frac{ln(\frac{{\lambda }_{ab}(T=0)}{{\xi }_{ab}(T=0)})+0.5}{{\lambda }_{ab}^{3}(T)}$$

where *μ*_0_ is the magnetic permeability of free space. Equation 25 is valid for thin superconductors if 2*b* < λ(0)^17,18,45–47^ and this should be the case for 1.0 nm thick MATBG.

Unfortunately, raw *B*_c2,⊥_(*T*) experimental data, from which to deduce ξ_ab_(0) for sample M2, is unavailable. However, it can be seen that ξ_ab_(0) for both sample M1 and sample M2 is practically identical if we consider values at optimal doping. This follows by inspection of *B*_c2,⊥_(*T*) in^7^ [Fig. 3e] for sample M1, where the comparison of *B*_c2,⊥_(*T*) for both samples is displayed in^7^ [Fig. 3a] and^7^ [Fig. 3b]. Thus, for the analysis of sample M2 by Eq. 24 we will use the value of ξ_ab_(0) = 61.4 nm deduced from sample M1. This assumption is also supported by a recent report from Lu *et al*.^11^ (in their Extended Data Fig. 3), who measured *B*_c2_(*T* = 16 mK) for a broad range of doping states for MATBG with *θ* ~ 1°. We analyse the Lu *et al*.^11^ data in the following section.

There are several recent first principle calculation papers where different types of superconducting gap symmetries are proposed to present in MATBG. For instance, *d−*, *p*−, and exotic *d* + *id-*wave symmetries, where *d* + *id-*wave symmetry was initially considered by Laughlin^48^ twenty years ago for HTS cuprates. We only mention^13,14,49–56^ where models and extended reference lists can be found.

However, of more importance is whether such gap symmetries are supported by experimental data. Therefore, we fit the available data using an extended BCS model with different expressions for the gap symmetry and compare the deduced parameters with weak-coupling BCS limits. Based on this we can infer which gap symmetries can potentially explain the measured behaviour. Here, we fit the experimental *J*_c_(sf,*T*) data for sample M2 to Eq. 25 by utilizing different expressions for the symmetry of the superconducting gap. For these expressions, we again draw from the approach proposed by Gross-Alltag *et al*.^28^ for *s*−, *d−*, and *p*-wave symmetries. For *d* + *id* symmetry we use an approach proposed by Pang *et al*.^57^.

Equations for λ(*T*) and Δ(*T*) for *s*-wave symmetry have already been presented in Eqs. 9, 10 respectively, and in Fig. 4(a) a fit of the *J*_c_(sf,*T*) data to single band *s*-wave model is shown. All the deduced parameters are presented in Table 1.

**Table 1:** Deduced parameters for *single-band* models applied to MATBG sample M2^7^ doped at *n*_n_ = −1.44 10^12^ cm^−2^ where an effective mass of charge carriers m*/*m*_e_ = 0.1637 ± 0.0154 was used, which was obtained from the analysis presented in Fig. 5.

Model	*T*_c_ (K)		Δ(0) (μeV)	2Δ(0)/k_B_*T*_c_	Δ*C*/*C*	*v*_F_ (10^4^ m/s)	*T*_F_ (K)	*T*_c_/*T*_F_	λ(0) (nm)
*s*-wave	1.28 ± 0.05		245 ± 9	4.4 ± 0.2	2.7 ±0.9	7.3 ± 0.3	28.6 ± 2.0	0.045 ± 0.002	2,182 ± 3
*d*-wave (low-*T* asymptote)			2,300 ± 500	>40					2,166 ± 5
*p*-wave polar **A**⊥***l***	1.24 ± 0.06		>10^6^	>10^4^	>7				2,142 ± 25
*p*-wave polar **A**||***l***	1.34 ± 0.05		430 ± 32	7.4 ± 0.6	1.0 ± 0.3	13 ± 1	86 ± 15	0.015 ± 0.003	2,180 ± 3
*p*-wave axial **A**⊥***l***	1.31 ± 0.05		301 ± 13	5.4 ± 0.2	1.9 ± 0.6	8.8 ± 0.3	42 ± 3	0.031 ± 0.002	2,183 ± 3
*p*-wave axial **A**||***l***	1.37 ± 0.05		1,500 ± 1,200	27 ± 22	2.3 ± 0.5	46	1,100	>0.001	2,178 ± 3
*d* + *id*-wave	1.27 ± 0.05	Gap 1	322 ± 38	5.92 ± 0.70	0.959 (fixed)	9.5 ± 1.0	49 ± 10	0.026 ± 0.003	2,180 ± 3
		Gap 2	187 ± 24	3.44 ± 0.30	0.959 (fixed)	5.5 ± 0.5	16 ± 3	0.078 ± 0.016	

For the case of *d* + *id* we fixed the ratio of Δ*C*/*C* = 0.995 for both gaps. The ground-state coherence length was assumed to be ξ(0) = 61.4 ± 1.7 nm.

Next, we performed a fit to a *d*-wave gap symmetry model. Using a 2D cylindrical Fermi surface the equation for the London penetration depth is as follows:

26 $$\lambda (T)=\frac{\lambda (0)}{\sqrt{1-\frac{1}{2\cdot {k}_{B}\cdot T}\cdot {\int }_{0}^{2\pi }co{s}^{2}(\theta )\cdot ({\int }_{0}^{\infty }\frac{d\varepsilon }{cos{h}^{2}(\frac{\sqrt{{\varepsilon }^{2}+{\Delta }^{2}(T,\theta )}}{2\cdot {k}_{B}\cdot T})})\cdot d\theta }}$$

where the superconducting energy gap, Δ(*T*, *θ*), is given by:

27 $$\Delta (T,\theta )={\Delta }_{m}(T)\cdot cos(2\theta )\,$$

where Δ_m_(*T*) is the is the maximum amplitude of the *k*-dependent *d*-wave gap given by Eq. 10, *θ* is the angle around the Fermi surface subtended at (*π*, *π*) in the Brillouin zone (details can be found elsewhere^58,59^). In Eq. 10 the value of *η* = 7/5^58^. The fit to this model does not converge, as the value Δ_m_(0) tends toward an infinitely large value.

A fit can still be obtained by using a low-*T* asymptote of the single-band *d*-wave model which is presented in Fig. S4 It can be seen (Fig. S4, Table 1) that the deduced Δ_m_(0) = 2.3 ± 0.5 meV is unacceptably large. These results indicate that phonon-mediated *d*-wave symmetry in MATBG is not supported by experimental data and should be omitted from further consideration.

Fitting a *p*-wave gap symmetry model is more complicated (compared with *s*− and *d*-wave) because in this case the gap function is given by^28^:

28 $$\Delta (\hat{{\boldsymbol{k}}},T)=\Delta (T)f(\hat{{\boldsymbol{k}}},\hat{{\boldsymbol{l}}})$$

where, Δ(*T*) is the superconducting gap amplitude, ***k*** is the wave vector, and ***l*** is the gap axis. The electromagnetic response depends on the mutual orientation of the vector potential and the gap axis. In an experiment this is given by the orientation of the crystallographic axes compared with the direction of the electric current. There are two different *p*-wave pairing states: “axial” where there are two point nodes, and “polar” where there is an equatorial line node. The shape of the London penetration depth, λ(*T*) for *p*-wave polar **A**||***l*** and axial **A**⊥***l*** cases are difficult to distinguish from the *s*-wave counterpart, and the *p*-wave axial **A**||***l*** case is difficult to distinguish from the dirty *d*-wave case^28,59^.

Despite these difficulties there is still a possibility to make a distinction based on the values of the deduced fundamental superconducting parameters, in particular by considering the ratios of $\frac{2\cdot \Delta (0)}{{k}_{B}\cdot {T}_{c}}$ and Δ*C*/*C*. These two ratios are given in Table 2 for *p*-wave and other gap symmetries^28^.

**Table 2:** BCS weak-coupling limit values for 2Δ(0)/*k*_B_*T*_c_ and Δ*C*/*C* for *s*−, *d−*, *p*−, and *d* + *id*-wave superconducting gap symmetries^28,59^.

Pairing symmetry and experiment geometry	$\frac{{\bf{2}}\cdot {\boldsymbol{\Delta }}({\bf{0}})}{{{\boldsymbol{k}}}_{{\boldsymbol{B}}}\cdot {{\boldsymbol{T}}}_{{\boldsymbol{c}}}}$ or $\frac{{\bf{2}}\cdot {{\boldsymbol{\Delta }}}_{{\boldsymbol{m}}}({\bf{0}})}{{{\boldsymbol{k}}}_{{\boldsymbol{B}}}\cdot {{\boldsymbol{T}}}_{{\boldsymbol{c}}}}$	$\frac{{\boldsymbol{\Delta }}{\boldsymbol{C}}}{{\boldsymbol{C}}}$
*s*-wave	3.53	1.43
*d*-wave	4.28	0.995
*p*-wave; polar **A**⊥***l***	4.924	0.792
*p*-wave; polar **A**||***l***	4.924	0.792
*p*-wave; axial **A**⊥***l***	4.058	1.188
*p*-wave; axial **A**||***l***	4.058	1.188
*d* + *id*	3.85	depends on the ratio of two gap amplitudes; $0.995\le \frac{\Delta C}{C}\le 1.43$

Δ_*m*_(0) is the maximum amplitude of the *k*-dependent *d*-wave gap, $\Delta (\theta )={\Delta }_{m}(0)\cdot \,\cos (2\theta )$.

A gap equation for the *p*-wave case was given by Gross-Alltag *et al*.^28^ which is identical to Eq. 10, but *η* is given by:

29 $${\eta }_{p,a}=\frac{2}{3}\cdot \frac{1}{{\int }_{0}^{1}\,{f}_{p,a}^{2}(x)\cdot dx}\,$$

where

30 $${f}_{p}(x)=x;\,{\rm{polar}}\,{\rm{configuration}}$$

31 $${f}_{a}(x)=\sqrt{1-{x}^{2}};\,{\rm{axial}}\,{\rm{configuration}}$$

This gap equation was substituted into the temperature dependent London penetration depth equation given also by Gross-Alltag *et al*.^28^:

32 $${\lambda }_{(p,a)(\perp ,\parallel )}(T)=\frac{{\lambda }_{(p,a)(\perp ,\parallel )}(0)}{\sqrt{1-\frac{3}{4\cdot {k}_{B}\cdot T}\cdot {\int }_{0}^{1}\,{w}_{\perp ,\parallel }(x)\cdot ({\int }_{0}^{\infty }\frac{d\varepsilon }{cos{h}^{2}(\frac{\sqrt{{\varepsilon }^{2}+{\Delta }_{p,a}^{2}(T)\cdot {f}_{p,a}^{2}(x)}}{2\cdot {k}_{B}\cdot T})})\cdot dx}}$$

where the function ${w}_{\perp ,\parallel }(x)$ is ${w}_{\perp }(x)=(1-{x}^{2})/2$ and ${w}_{\parallel }(x)={x}^{2}$.

By substituting Eqs. 10, 29–32 in Eq. 25, one can fit the experimental *J*_c_(sf,*T*) data to the polar and axial *p*-wave model and deduce λ(0), Δ(0), Δ*C*/*C* and *T*_c_ as free-fitting parameters.

The result for only one *p*-wave configuration (axial *A*⊥*l*) is shown in Fig. 4(b) as this has the only meaningfully deduced parameters. The other three fits are presented in Fig. S5. The deduced parameters for all cases are presented in Table 1.

The gap equation for the case of *d* + *id* gap symmetry is considered elsewhere^48,49,53,56^. In this paper we use the approach proposed by Pang *et al*.^57^:

33 $$\begin{array}{rcl}\Delta (T,\theta ) & = & {[{({\Delta }_{1}(0)\cdot cos(2\cdot \theta ))}^{2}+{({\Delta }_{2}(0)\cdot sin(2\cdot \theta ))}^{2}]}^{1/2}\\  &  & \cdot \,(tanh[\frac{2\cdot \pi }{{\alpha }_{{\rm{BCS}},d+id-{\rm{wave}}}}\cdot \sqrt{\eta \cdot \frac{\Delta C}{C}\cdot (\frac{{T}_{c}}{T}-1)}])\end{array},$$

where Δ_1_(0) and Δ_2_(0) are the two *d*-wave gap amplitudes, *η* = 7/5, and *θ* is the angle around the Fermi surface subtended at (*π*, *π*) in the Brillouin zone, and *α*_BCS,d+id-wave_ is the double band gap ratio (details can be found elsewhere^56,57^).

Because the experimental *J*_c_(sf,*T*) dataset was not dense and the two gap model has many parameters, we were forced to reduce the number of free-fitting parameters so as not to overfit the data. We therefore have chosen to fix:

1. Δ*C*/*C* was set to the weak-coupling limit of BCS theory for *d*-wave superconductors, Δ*C*/*C* = 0.959.
2. The double band gap ratio ${\alpha }_{{\rm{BCS}},d+id-{\rm{wave}}}$ was set to its weak-coupling limit which is 1.925.

The fit values obtained for this *d* + *id* model (Table 1) give the two values of the gap ratios:

34 $$\frac{2\cdot {\Delta }_{1}(0)}{{k}_{B}\cdot {T}_{c}}=5.92\pm 0.70,$$

35 $$\frac{2\cdot {\Delta }_{2}(0)}{{k}_{B}\cdot {T}_{c}}=3.44\pm 0.30.$$

where 2∙Δ_1_ (0)/*k*_B_*T*_c_ far exceeds the weak coupling limit of 3.85 for *d* + *id* symmetry superconductor^48^. This therefore suggests that *d* + *id* symmetry should also be excluded from further consideration.

The analysis of the self-field critical current density in MATBG within a single band model shows that:

1. MATBG has an equal chance to be moderately strong coupled *s*-wave or *p*-wave superconductor.
2. The ground-state London penetration depth is independent of gap symmetry:
  36 $$\lambda (0)=2,\,183\pm 3\,nm.$$
3. The GL parameter κ(0) is:

37 $$\kappa (0)=\frac{\lambda (0)}{\xi (0)}=35.6.$$

The Fermi velocity *v*_ab,F_ and the Fermi temperature *T*_F_ for the sample M2 (*θ* = 1.05°) based on the deduced values of ξ_ab_(0) and Δ(0) for all gap symmetries have been calculated using Eqs. 17 and 18 respectively, values are presented in Table 1.

**Figure 5:**
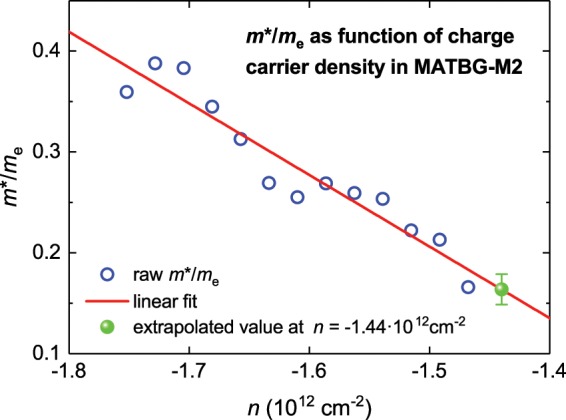
Raw experimental m*(*n*)/*m*_e_ data with a linear fit where the goodness of fit was *R* = 0.911. The green spot indicates the extrapolated m*(*n*)/*m*_e_ at *n* = −1.44 · 10^12^ cm^−2^.

This is done for MATBG at the doping state of *n* = −1.440 · 10^12^ cm^−2^. To do this, the experimental m*(*n*)/*m*_e_ data presented in of^7^ [Fig. 5e] is linearly extrapolated:

38 $${m}^{\ast }(n=-\,1.440\cdot {10}^{12}\,c{m}^{-2})=(0.1637\pm 0.0154)\cdot {m}_{e},$$

which is shown in Fig. 5. This value is close to the last experimental data point in^7^ [Fig. 5e]:

39 $${m}^{\ast }(n=-\,1.468\cdot {10}^{12}\,c{m}^{-2})=0.1653\cdot {m}_{e}.$$

All the calculated *T*_F_ values are in presented in Table 3 together with the ratio *T*_c_/*T*_F_. This ratio varied in the range:

40 $$0.031\le \frac{{T}_{c}}{{T}_{F}}\le 0.045,$$

with the lower limit corresponding to the *p*-wave fit and upper limit corresponding to the *s*-wave fit. It should be noted that both lower and upper limits of this ratio are within the range (Eq. 22) established for all unconventional superconductors by Uemura *et al*.^30,31^.

**Table 3:** Deduced parameters for *two-band* models for MATBG sample M2 (ref. ^7^) doped at *n*_n_ = −1.44   10^12^ cm^−2^ where for both bands we assumed an effective mass of charge carriers, m*/*m*_e_ = 0.1637 ± 0.0154, and κ_c_ = 35.6 (Eq. 37).

Model		*T*_c_ (K)	Δ(0) (μeV)	2Δ(0)/k_B_*T*_c_	Δ*C*/*C*	*v*_F_ (10^4^ m/s)	*T*_F_ (K)	*T*_F_/*T*_c_	λ(0) (nm)	$\frac{{{\boldsymbol{\rho }}}_{{\boldsymbol{s}},{\boldsymbol{band}}{\bf{2}}}}{{{\rho }}_{{\boldsymbol{s}},{\boldsymbol{band}}{\bf{1}}}}$
Two-band *s*-wave	Gap 1	1.42 ± 0.10	300 ± 54	4.9 ± 0.8	1.3 ± 0.7	8.8 ± 1.5	42 ± 14	0.034 ± 0.008	2,209 ± 17	0.13 ± 0.04
	Gap 2	0.5 ± 0.2	84 ± 28	4.0 ± 1.5		6.8 ± 2.3	25 ± 14	0.02 ± 0.01	6,125 ± 1057	
Two-band axial **A**⊥***l**** p*-wave	Gap 1	1.44 ± 0.12	390 ± 109	6.3 ± 2.0	0.9 ± 0.5	11.4 ± 2.8	70 ± 35	0.02 ± 0.01	2,204 ± 18	0.11 ± 0.04
	Gap 2	0.5 ± 0.2	98 ± 48	4.5 ± 2.5		7.2 ± 3.5	28 ± 19	0.02 ± 0.01	6,467 ± 1385	

From the deduced λ(0) values (Eq. 36) the Cooper pair density, *n*_s,C_, in MATBG (*θ* = 1.05°) can be calculated giving:

41 $${n}_{s,C}=\frac{1}{2}\cdot \frac{{m}^{\ast }}{{\mu }_{0}\cdot {e}^{2}\cdot {\lambda }^{2}(0)}=(4.9\pm 0.4)\cdot {10}^{23}\,{m}^{-3}.$$

By using a thickness of 2*b* = 1.0 nm, the surface pair density is:

42 $${n}_{s,C,surf}=\frac{1}{2}\cdot \frac{{m}^{\ast }}{{\mu }_{0}\cdot {e}^{2}\cdot {\lambda }^{2}(0)}\cdot 2b=(4.9\pm 0.4)\cdot {10}^{14}\,{m}^{-2}=(4.9\pm 0.4)\cdot {10}^{10}\,c{m}^{-2}$$

From Eqs. 41, 42 the ratio of the Cooper pair density to the total carrier density ${n}_{n}=-\,1.44\cdot {10}^{12}\,c{m}^{-2}$ is calculated to be

43 $$\frac{{n}_{s,C,surf}}{{n}_{n}}=0.034\pm 0.003.$$

This value is also in the same range as other unconventional superconductors^30,31^.

### Self-field critical current analysis: two-superconducting band models

Now we can consider the question: does the available experimental *J*_c_(sf,*T*) data support the existence of two-band superconductivity in MATBG, where one band originates from intralayer coupling, and the second from a weak van der Waals interlayer interaction. We fit the *J*_c_(sf,*T*) data to a two-band model proposed earlier^17^:

44 $${J}_{c}(sf,T)={J}_{c,band1}(sf,T)+{J}_{c,band2}(sf,T)$$

where each band is described by Eq. 25. Because each band has four free-fitting parameters, i.e. λ(0), Δ(0), Δ*C*/*C* and *T*_c_, we were forced to reduce the number of parameters, and as we found in^17^, the most appropriate approach is to equalize Δ*C*/*C* for both bands, i.e.:

45 $$\frac{\Delta {C}_{1}}{{C}_{1}}=\frac{\Delta {C}_{2}}{{C}_{2}}$$

**Figure 6:**
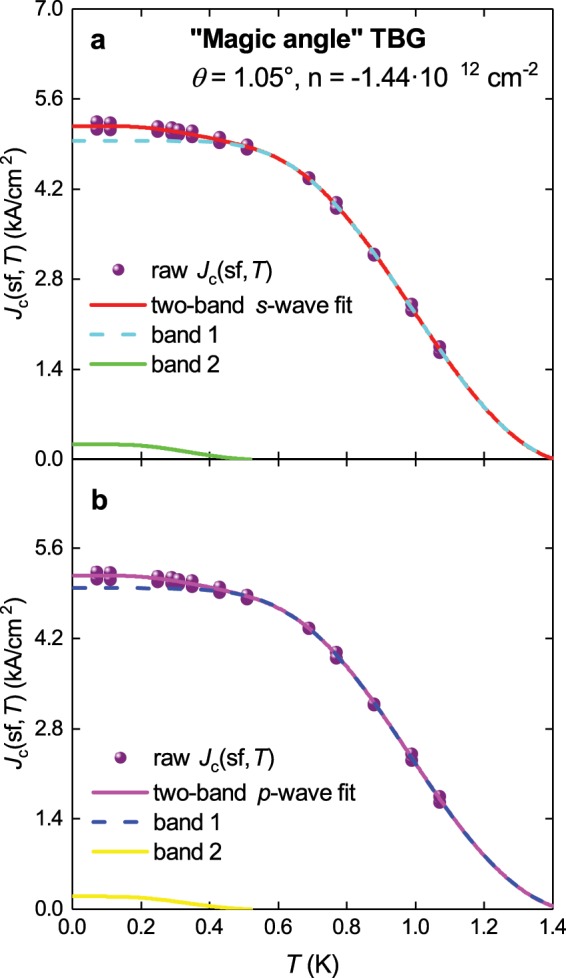
The self-field critical current density, *J*_c_(sf,*T*), for sample M2 (*θ* = 1.05°) from the work of Cao et al.^7^ and a fit of the data to *s*- and *p*-wave two-band models. For both models and both bands κ_c_(0) = 35.6 (Eq. 36) was used. (**a**) *s*-wave fit, the goodness of fit *R* = 0.9965; (**b**) *p*-wave axial **A**⊥***l*** fit, *R* = 0.9965. Deduced parameters are in Table 3.

We also assume that for both bands κ_c_ = 35.6 (Eq. 37). The result of fits to two-band *s*-wave and two-band *p*-wave axial **A**⊥***l*** models are shown in Fig. 6(a,b), respectively. There is experimental evidence that at *T* ~ 0.5 K a new superconducting band opens. The deduced parameters for both fits are in Table 3.

It can be seen from Fig. 6 and Table 3, that there is not a significant differences between transition temperatures, *T*_c1_ and *T*_c2_, of each model. nor is there a significant difference comparing the difference between the BCS ratios of $\frac{2\cdot {\Delta }_{1}(0)}{{k}_{B}\cdot {T}_{c1}}$ and $\frac{2\cdot {\Delta }_{2}(0)}{{k}_{B}\cdot {T}_{c2}}$ for both bands in both models.

The main difference is in the superfluid densities of the bands, i.e., ${\rho }_{s,1}\equiv \frac{1}{{\lambda }_{1}^{2}(0)}$ and ${\rho }_{s,2}\equiv \frac{1}{{\lambda }_{2}^{2}(0)}$, which are different by an order of magnitude. This is a reasonable result given the interlayer charge carrier concentration is much lower in comparison with interlayer one, as the two SGL are only interacting via weak van der Waals forces.

### MATBG phase diagram

Here we use Eq. 24 to analyse data reported by Lu *et al*.^11^ on the phase diagram of MATBG with θ ~ 1° measured for a wide range of doping states. We note that Lu *et al*.^11^ clearly observe at least six superconducting domes shown in^11^ [Fig. 1d]. We analyse *B*_c2,_(*T* = 16 mK) and *I*_c_(sf, *T* = 16 mK) data for the sample D1 which is displayed in Extended Data^11^ [Fig. 3a] and Extended Data^11^ [Fig. 6j–m] respectively.

**Figure 7:**
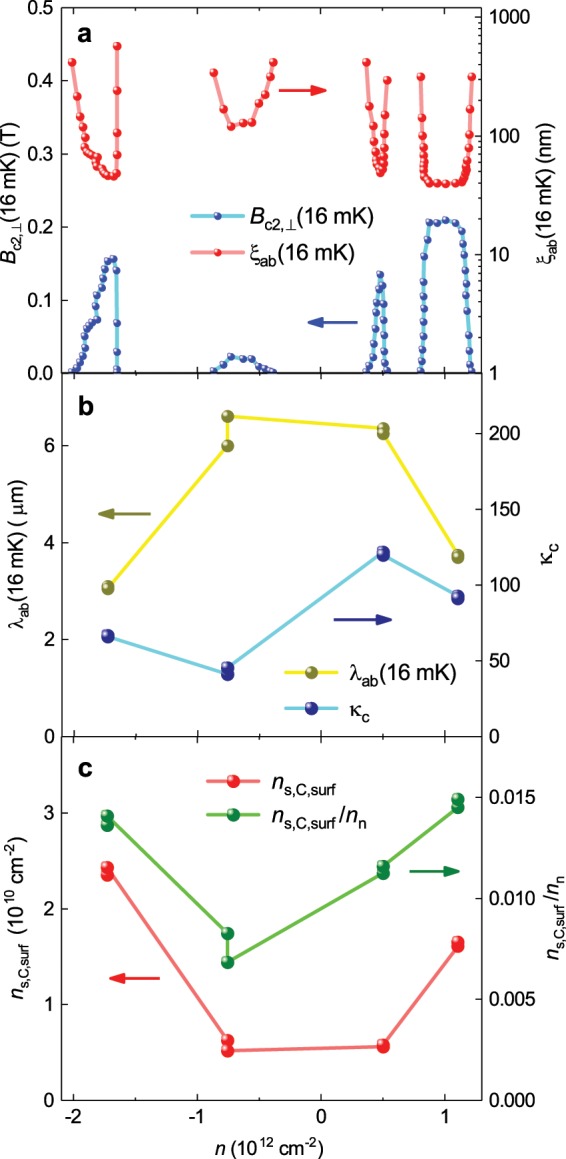
Analysis of the superconducting phase diagram of MATBG with θ ~ 1.1°. (**a**) The upper critical field, *B*_c2,⊥_(16 mK), and deduced ξ_ab_(16 mK) by Eq. 1. (**b**) Deduced λ_ab_(16 mK) and κ_c_ for four doping states for which *I*_c_(sf, 16 mK) was reported by Lu *et al*.^11^. (**c**) Cooper pairs surface density, ${n}_{s,C,surf}$, and the ratio of $\frac{{n}_{s,C,surf}}{{n}_{n}}$ for four doping states for which *I*_c_(sf, 16 mK) was reported by Lu *et al*.^11^.

By using a sample D1 width, 2*a* = 2 μm, and thickness, 2*b* = 1 nm, we calculate *J*_c_(sf, *T* = 16 mK) for four doping states where *I*_c_(sf, *T* = 16 mK) data was reported. By using the deduced ξ_ab_(*T* = 16 mK) for the same doping state we numerically solved Eq. 24 and deduced λ_ab_(*T* = 16 mK), κ_c_(*T* = 16 mK), ${n}_{s,C,surf}$, and $\frac{{n}_{s,C,surf}}{{n}_{n}}$ (Fig. 7).

An interesting result is that for all four different superconducting domes, the ratio of:

46 $$0.007\le \frac{{n}_{s,C,surf}}{{n}_{n}}\le 0.015\,$$

does not change significantly over the whole phase diagram. What is more surprizing is that two superconducting domes located near *n*_n_ = −1.73 10^12^ cm^−2^ and *n*_n_ = 1.11 10^12^ cm^−2^ have practically the same $\frac{{n}_{s,C,surf}}{{n}_{n}}\cong 0.015$, and more or less close values for λ_ab_(*T* = 16 mK) and κ_c_(*T* = 16 mK).

## Conclusions

In this paper we use existing BCS and GL models to analyze raw experimental data from MATBG reported by Cao *et al*.^7^ and Lu *et al*.^11^. Surprisingly enough, the results of our analysis show that MATBG has a very low charge carrier effective mass, ${m}^{\ast }=(0.164\pm 0.015)\cdot {m}_{e}$, and is located in the Uemura plot (Fig. 2), next to the heavy fermion superconductors^1^, particularly to UBe_13_ which has ${m}^{\ast } \sim 200\cdot {m}_{e}$ ^2^. This places MATBG in the same band of *T*_F_/*T*_c_ values as all other unconventional superconductors as categorized by Uemura, contrary to^7^ [Fig. 6] which shows MATBG approaching the Bose-Einstein condensation line.

Our analysis of the temperature dependent upper critical field and the self-field critical current experimental data show that three of four *p-*wave as well as *d−*, and *d* + *id*-wave symmetries should be excluded from further consideration as possible phonon-electron mediated pairing symmetries in MATBG. Furthermore, the analysis indicates that, when viewed in the established phenomenology of superconductivity, MATBG is a *moderately strong coupled two-band superconductor* with *s*- or *p*-wave symmetry. Although, further experimental data is needed to differentiate the two remaining pairing symmetries. Because graphene has planar honeycomb lattice of *sp*^2^ bonded carbon atoms, our findings of either *s*- or *p*-wave pairing symmetry have supporting background evidence.

## Supplementary information

SUPPLEMENTARY INFORMATION.
