# Supplementary material for: Classifying superconductivity in Moiré graphene superlattices
Source: Sci Rep. 2020 Jan 14;10:212. doi: 10.1038/s41598-019-57055-w (PMC6959361; doi:10.1038/s41598-019-57055-w)
Supplement: Supplementary file 1 — SUPPLEMENTARY INFORMATION. [file 41598_2019_57055_MOESM1_ESM.pdf]

SUPPLEMENTARY INFORMATION

Classifying superconductivity in Moiré graphene superlattices

E.F. Talantsev[1,2]*, R.C. Mataira[3] and W.P. Crump[4,5]

[1]M.N. Mikheev Institute of Metal Physics, Ural Branch, Russian Academy of Sciences, 18, S. Kovalevskoy St., Ekaterinburg, 620108, Russia

[2]NANOTECH Centre, Ural Federal University, 19 Mira St., Ekaterinburg, 620002, Russia

[3]Robinson Research Institute, University of Wellington, 69 Gracefield Road, Lower Hutt, 5040, New Zealand

[4]MacDiarmid Institute for Advanced Materials and Nanotechnology, P.O. Box 33436, Lower Hutt 5046, New Zealand

[5]Aalto University, Foundation sr, PO Box 11000, FI-00076 AALTO, Finland

*E-mail: evgeny.talantsev@imp.uran.ru

Supplementary Table S1. Deduced superconducting parameters for MATBG sample M1.

Model	$\xi_{ab}(0)$ (nm)	T_c (K)
Gorter-Casimir [S1,S2]	63.7 ± 0.7	0.488 ± 0.004
WHH [S3,S4]	60.6 ± 1.0	0.503 ± 0.007
B-WHH [S5]	59.7 ± 0.4	0.497 ± 0.003
JHC [S6]	58.3 ± 0.6	0.515 ± 0.005
Gor’kov [S7]	61.9 ± 0.4	0.494 ± 0.003
Eq. 18 [S8]	64.0 ± 0.8	0.504 ± 0.007
Average	61.4 ± 1.7	0.500 ± 0.006

Supplementary Table S2. Deduced $I_c(\mathrm{sf},T)$ from fit of $V(I)$ curves to Eq. 23.

Temperature (K)	$I_c(\mathrm{sf},T)$ (nA)	Uncertainty in deduced $I_c(\mathrm{sf},T)$ (nA)	*n-value* in Eq. 1	Uncertainty in deduced *n-value*
0.07	53.9	<0.01	48.2	<2
0.07	55.0	<0.01	61.6	<2
0.11	54.9	<0.01	58.8	<2
0.11	53.8	<0.01	45.4	<2
0.25	53.4	<0.01	45.3	<2
0.25	54.3	<0.01	58	<2
0.29	53.1	<0.01	45	<2
0.29	54.1	<0.01	56.9	<2
0.31	52.9	<0.01	52	<2
0.31	53.8	<0.01	51.1	<2
0.35	52.6	<0.01	38	<2
0.35	53.5	<0.01	43.6	<2
0.43	51.7	<0.01	45.2	<2
0.43	52.5	<0.01	43	<2
0.51	50.6	0.01	31.9	0.3
0.51	51.2	0.01	37.9	0.9
0.69	45.7	0.06	16	0.4
0.69	45.9	0.08	17.3	0.4
0.77	41.9	0.06	11.1	0.2
0.77	41	0.1	10.6	0.4
0.88	33.0	0.3	5.4	0.2
0.88	33.5	0.3	5.5	0.2
0.99	24.3	1	2.8	0.2
0.99	25.3	2	3.2	0.2
1.07	17.4	2	1.9	0.2
1.07	18.4	2	2	0.2
1.26	7	31	1.1	0.5
1.26	7	47	1.1	0.7

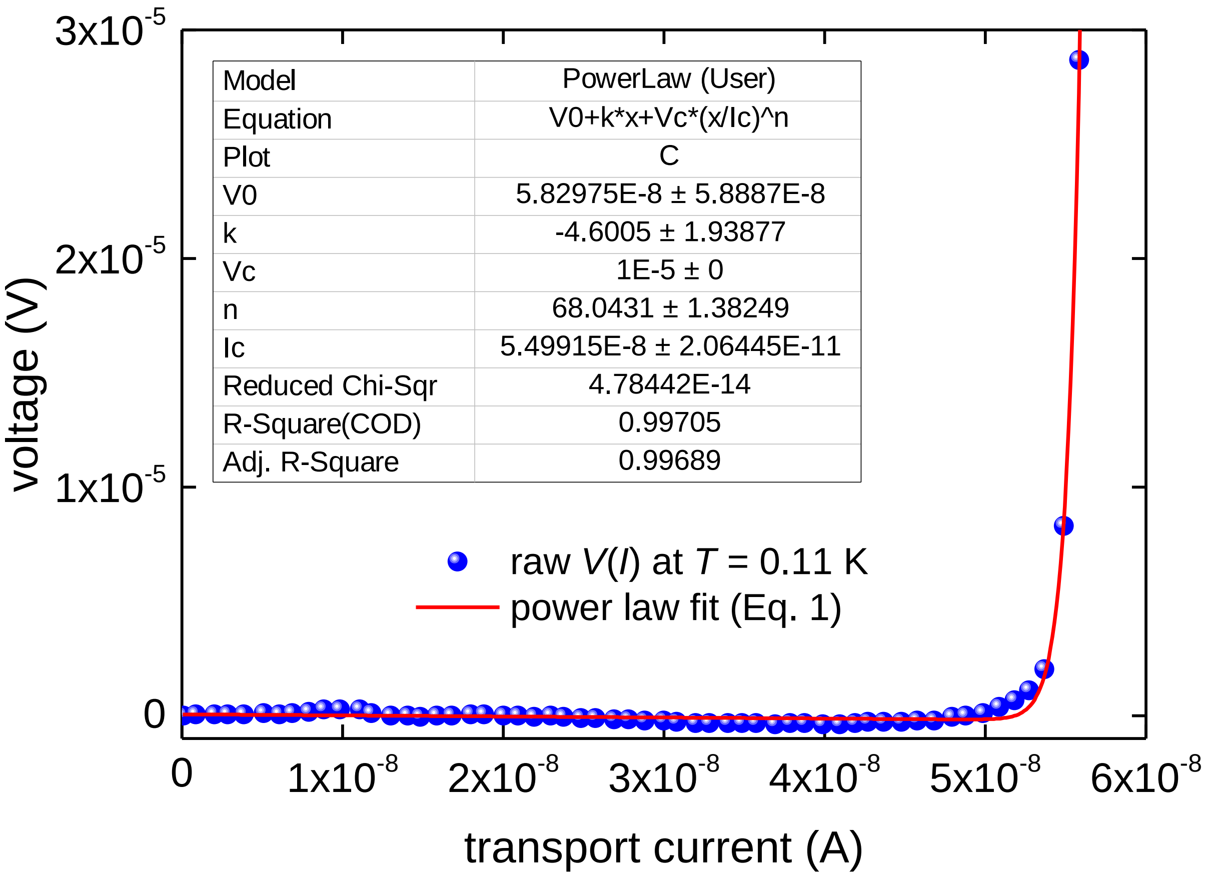

Supplementary Figure S1. Raw $V(I)$ curve at T = 0.11 K and data fit to Eq. 22.

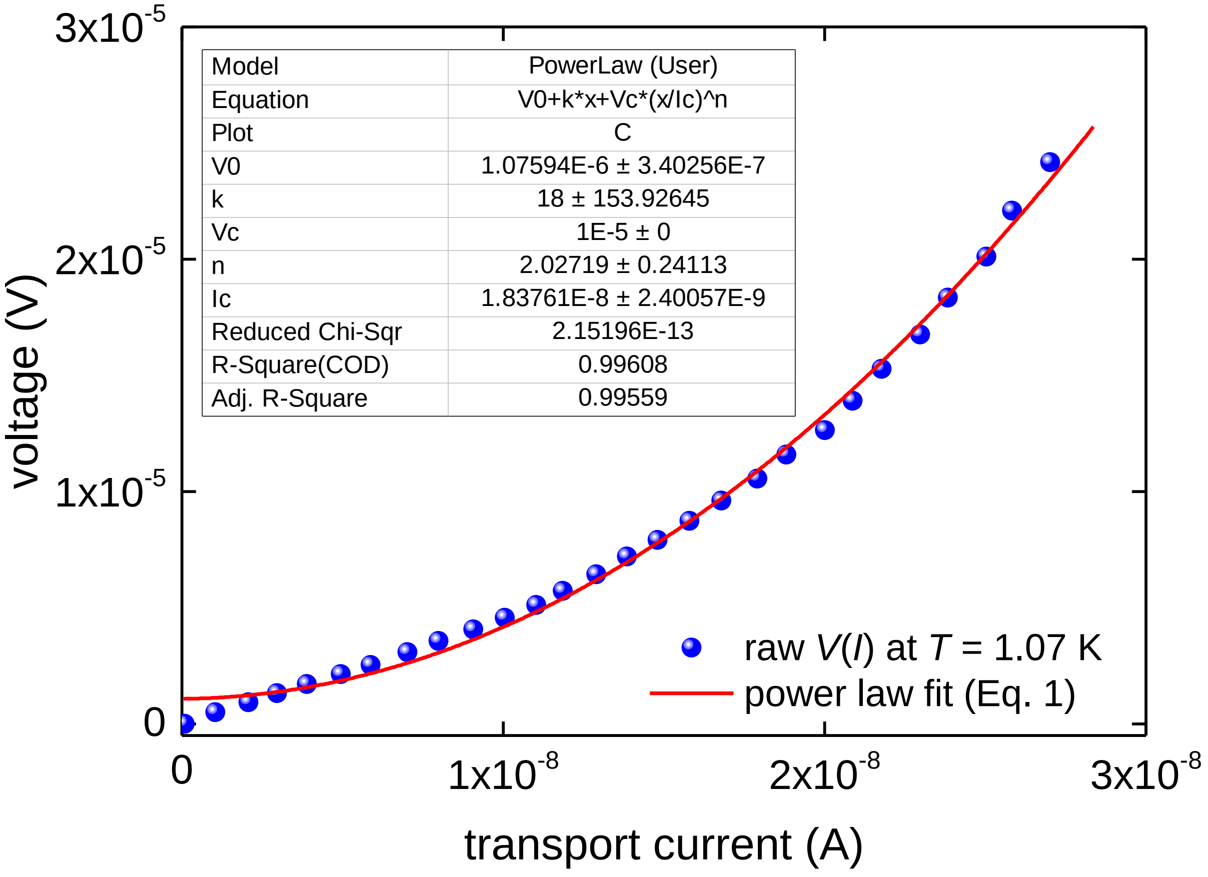

Supplementary Figure S2. Raw $V(I)$ curve at T = 1.07 K and data fit to Eq. 22.

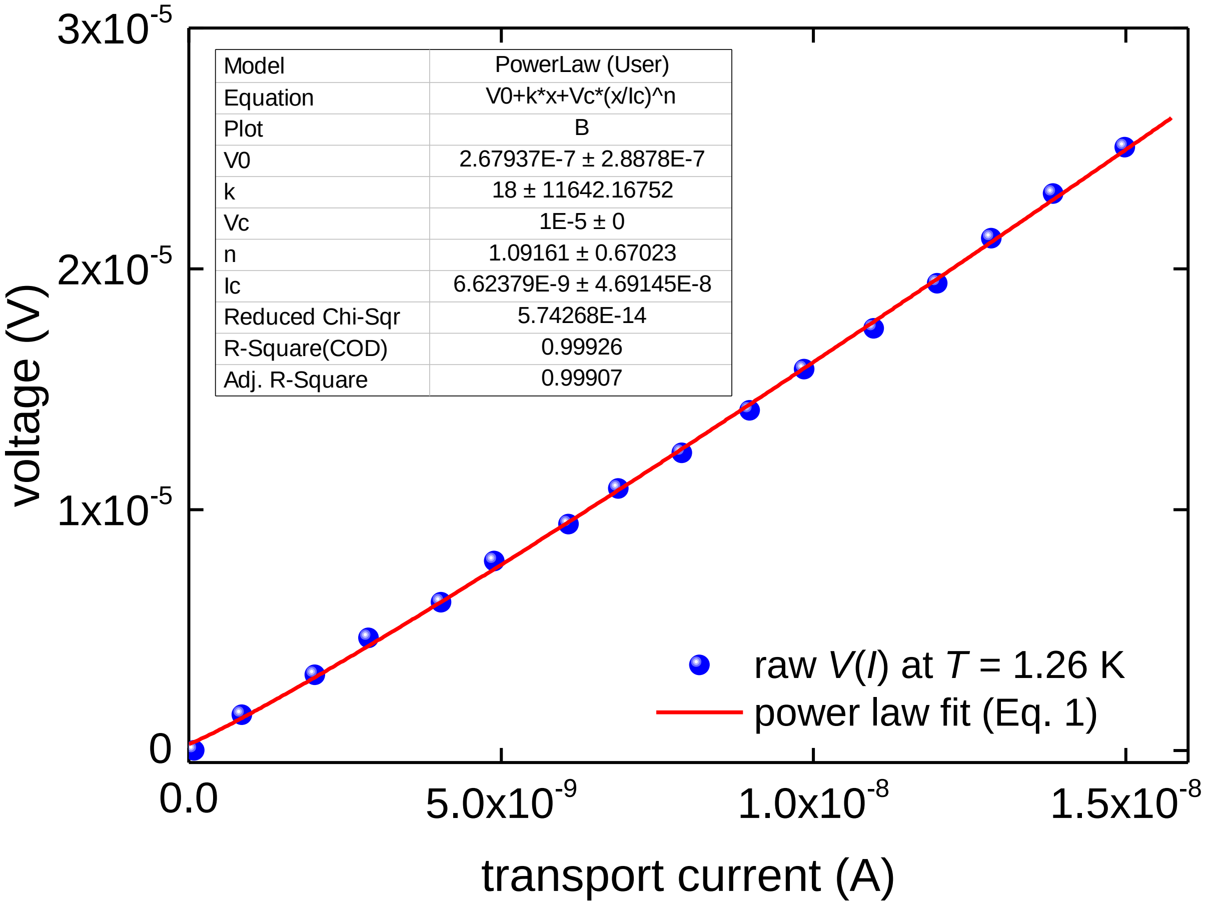

Supplementary Figure S3. Raw $V(I)$ curve at T = 1.26 K and data fit to Eq. 22.

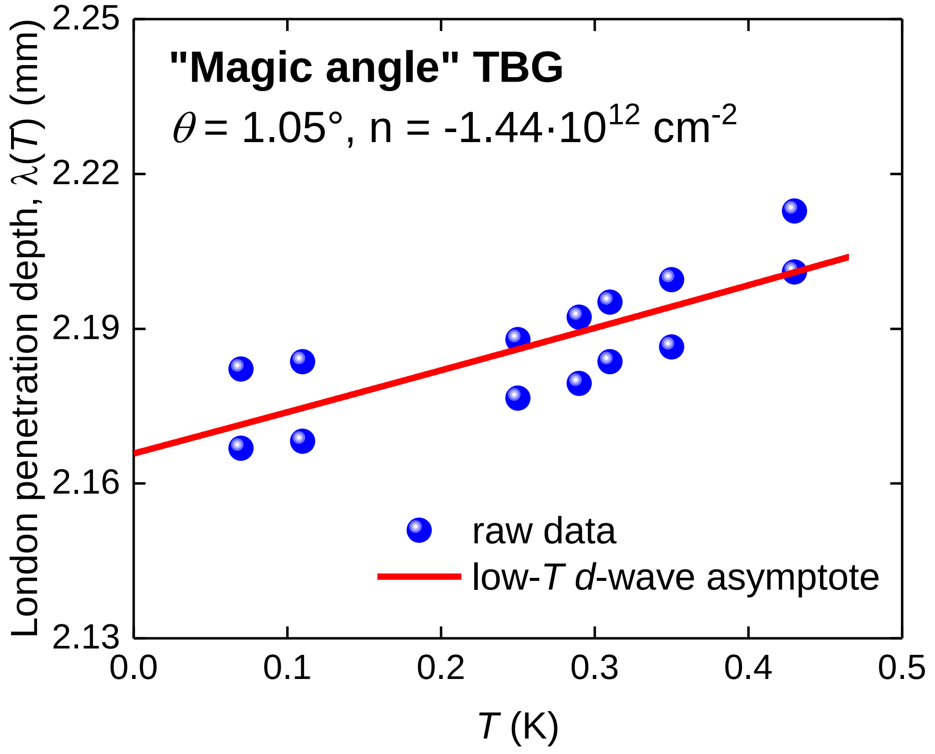

Supplementary Figure S4. London penetration depth, $\lambda(T)$, for sample M2 ($\theta = 1.05°$) of work of Cao et al [7] and data fit to low-temperature asymptote of *d*-wave model (see details in main paper and Table III). For this model we used $\kappa = 35.6$. The goodness of fit R = 0.6352.

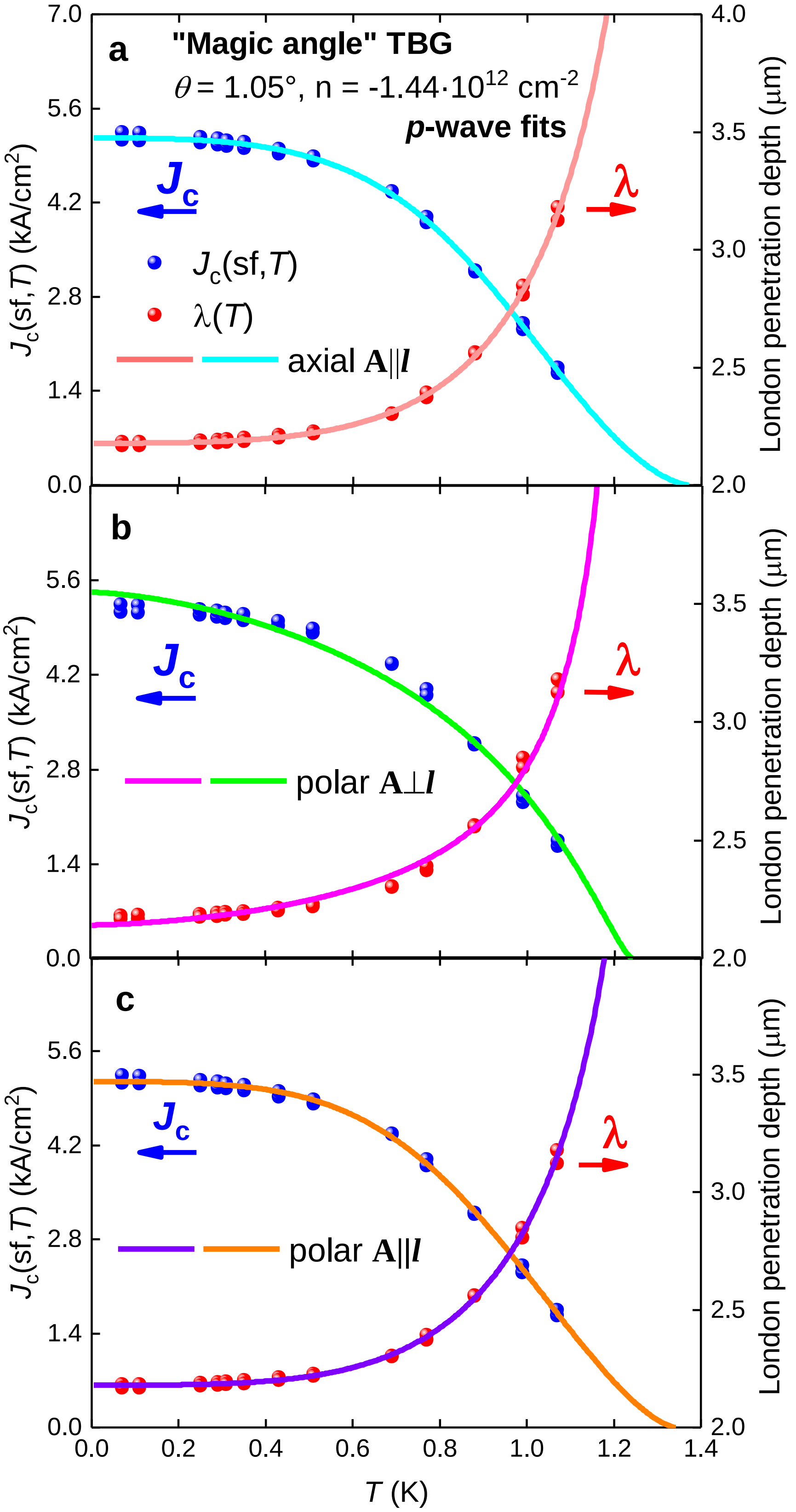

Supplementary Figure S5. The self-field critical current density, $J_c(\mathrm{sf},T)$, for sample M2 (θ = 1.05°) of work of Cao et al [7] and data fit to three cases of *p*-wave model (see details in main paper and Table III). For all models we used κ = 35.6. (a) axial **A**||***l*** fit, the goodness of fit R = 0.9979; (b) *p*-wave polar **A**⊥***l*** fit, R = 0.9800; (c) polar **A**||***l*** fit, R = 0.9977.

Supplementary References

S1. Gorter, C. J., Casimir, H. On supraconductivity I. *Physica* **1**, 306-320 (1934).
S2. Poole, Ch. P. Jr., Creswick, R. J., Farach, H. A. and Prozorov, R. Superconductivity. (Elsevier, UK, Second edition, 2007).
S3. Helfand, E., Werthamer, N. R. Temperature and purity dependence of the superconducting critical field, H_{c2}. II. *Phys. Rev.* **147**, 288-294 (1966).
S4. Werthamer, N. R., Helfand, E. and Hohenberg, P. C. Temperature and purity dependence of the superconducting critical field, H_{c2}. III. Electron spin and spin-orbit effects *Phys. Rev.* **147**, 295-302 (1966).
S5. Baumgartner, T., Eisterer, M., Weber, H. W., Fluekiger, R., Scheuerlein, C., Bottura, L. Effects of neutron irradiation on pinning force scaling in state-of-the-art Nb_3Sn wires *Supercond. Sci. Technol.* **27**, 015005 (2014).
S6. Jones, C. K., Hulm, J. K. and Chandrasekhar, B. S. Upper critical field of solid solution alloys of the transition elements. *Rev. Mod. Phys.* **36**, 74-76 (1964).
S7. Gor'kov, L. P. The critical supercooling field in superconductivity theory. *Soviet Physics JETP* **10** 593-599 (1960).
S8. Talantsev, E. F. Classifying superconductivity in compressed H_3S. Modern Physics Letters B 33 1950195 (2019).
